# Notes on the Treatment of Diabetes Mellitus

**Published:** 1893-08-19

**Authors:** 


### NOTES ON THE TREATMENT OF DIABETES MELLITUS.

*The Hygienic and Dietetic Treatment.*—The hygienic treatment consists mainly in the avoidance of cold and damp. The clothing must be warm, and flannel should be worn next the skin. Regular and moderate exercise may be taken, but all fatigue must be most carefully guarded against. A warm bath, taken once or twice a week, is a useful and beneficial hygienic measure. Thirst may be alleviated by the use of acidulated drinks, of which dilute phosphoric acid is the most efficacious. Great relief is also often obtained by rinsing the mouth out with ice cold water, or by sucking small pieces of ice.

Constipation may be treated with castor oil or some mild laxative free from sugar. The purgative mineral waters often give good results. All active purgation must be carefully avoided.

The regulation of the diet is the main and all-important factor in the treatment of diabetes. The object to be attained is to give as great a variety of food as possible, while prohibiting the use of starch, and cane or grape sugar, and the substances which contain them.

Almost all kinds of animal food may be eaten by patients suffering from diabetes. Consequently, flesh, fish, and fowl are allowable; but liver and edible molluscs, such as mussels, cockles, &c., must be forbidden.

Cream, cheese, milk, butter, and eggs may all be included in the dietary of a diabetic. Milk, on theoretical grounds, is objectionable, but at St. Mary's it is generally included in the diet scale. Oil, especially in the form of cod liver oil, is often exceedingly useful. Soups and jellies may be used if no starch or sugar is used in the preparation of them.

Green vegetables, such as spinach, turnip or celery tops, lettuces, watercress, and green artichokes may be eaten. The green parts of asparagus and cabbage, if boiled in large quantities of water, may also be permitted. Most vegetables, however, are very harmful.

Potatoes, peas, beans, carrots, turnips, parsnips, sprouts, broccoli, sea kale, jerusalem artichoke, and beetroot are all to be entirely avoided. Sweet fruits are generally considered to be injurious, but nuts, if they can be digested, are harmless.

Flour of all kinds is injurious, and consequently bread must be prohibited. Rice, sago, arrowroot, macaroni, and vermicelli must also be entirely forbidden. One of the greatest difficulties in the dieting of a diabetic is to find an efficient substitute for bread. Several preparations have been devised, such as gluten bread or gluten cakes, bran cakes, and almond cakes, and these may all be tried, but most of them, on account of their costliness, and the large quantities in which they are required, are hardly suitable for hospital patients. Moreover, the patient frequently soon tires of all these preparations. At St. Mary's Hospital thin slices of bread, toasted perfectly hard and dry, are, as a rule, allowed.

Sugar and honey must, of course, be entirely eschewed.

Saccharine may be used instead of sugar as a sweetening agent, but it is not a very satisfactory substitute, as after its use the patient often complains that everything tastes sweet. Glycerine in small quantities, or mannite may also be used instead of sugar.

Tea and coffee, and cocoa made from nibs, may be drunk unsweetened. Dry sherry, claret, burgundy, and whiskey may be used in moderation, but champagne, port, cider, porter, brandy, rum, and gin must be entirely avoided. A little bitter beer, soda water, and the mineral waters may be allowed. Ginger-beer and lemonade are very objectionable. Diabetic patients should be abstemious in the use of alcohol in any form. Spirits are probably best taken as whiskey.

Before commencing the dietetic treatment of a diabetic patient it is usual to have him under observation for a few days, while he is taking ordinary food. During that time he should be weighed and the quantity and character of the urine should be "charted" daily. The diet should then be gradually restricted, and the effect on the weight of the patient and on urine should be noted every day. The daily analysis of the urine, with the quantity passed, should be arranged on a card kept for the purpose. The change to a strictly anti-diabetic diet should not be made too suddenly on account of the possible danger of the supervention of coma.

As soon as the effects of the restricted diet are clearly indicated the medical treatment should be commenced.

*The Medicinal Treatment.*—The most important drug in the treatment of diabetes is opium. It is most advantageously administered by the mouth, and may be given in the form of morphine or codeia. The latter preparation is the one more generally used, as it is supposed to cause less disturbance of digestion than the former. Codeia may at first be given in half grain doses three times a day, and this quantity may be gradually increased. As much as ten or twenty grains have been given in the twenty-four hours with beneficial effects. Patients suffering from diabetes show a marked tolerance of opium and its preparations.

In addition to the treatment by opium, it is of great importance to attend to the general health in cases of diabetes. For this purpose the administration of such drugs as iron, strychnine, quinine, and especially cod liver oil, will be found of considerable service.

The complications of diabetes should be treated on general principles. Operative procedure on patients suffering from diabetes are attended with danger. Should coma complicate the case, transfusion of blood, or the intra-venous injection of saline solution may be tried.

